# Complexity measure in natural time analysis identifying the accumulation of stresses before major earthquakes

**DOI:** 10.1038/s41598-024-81547-z

**Published:** 2024-12-28

**Authors:** Panayiotis A. Varotsos, Nicholas V. Sarlis, Toshiyasu Nagao

**Affiliations:** 1 Section of Condensed Matter Physics, Department of Physics, National and Kapodistrian University of Athens, Panepistimiopolis, Zografos, 157 84 Athens Greece; 2 Solid Earth Physics Institute, Department of Physics, National and Kapodistrian University of Athens, Panepistimiopolis, Zografos, 157 84 Athens Greece; 3 Institute of Oceanic Research and Development, Tokai University, 3-20-1, Orido, Shimizu-ku, Shizuoka, 424-0902 Japan

**Keywords:** Entropy, Time-series, Earthquakes, Complexity, Geophysics, Natural hazards, Statistical physics, thermodynamics and nonlinear dynamics

## Abstract

Here, we suggest a procedure through which one can identify when the accumulation of stresses before major earthquakes (EQs) (of magnitude *M* 8.2 or larger) occurs. Analyzing the seismicity in natural time, which is a new concept of time, we study the evolution of the fluctuations of the entropy change of seismicity under time reversal for various scales of different length *i* (number of events). Although the stress might be accumulating throughout the entire process of EQ preparation due to tectonic loading, here we find that the proposed complexity measure reveals different stress accumulation characteristics from those in the long-term background when the system approaches the critical stage. Specifically, we find that anomalous intersections between scales of different *i* are observed upon approaching a major EQ occurrence. The investigation is presented for the seismicity in Japan since 1984 including the *M*9 Tohoku EQ on 11 March 2011, which is the largest EQ ever recorded there, as well as for the seismicity before 2017 Chiapas M8.2 EQ, which is Mexico’s largest EQ in more than a century. Based on this new complexity measure, a preprint submitted on 5 December 2023 anticipated the 1 January 2024 M7.6 EQ in Japan.

## Introduction

It is widely known^1–3^ that earthquake (EQ) occurrences exhibit complex correlations in time, space and magnitude (e.g.,^4–9^) and the observed EQ scaling laws^10^ indicate the existence of phenomena closely associated with the proximity of the system to a critical point. In the 1980s, the observation of Seismic Electric Signals (SES), which are low frequency transient changes of the electric field of the Earth preceding EQs, was reported^11–13^. Many SESs observed within a short time are termed SES activity^14^ being accompanied by Earth’s magnetic field variations^15^ mainly on the z-component^16,17^. These observations have been motivated by a physical model for SES generation, which enables the explanation of the simultaneous detection of additional transient multidisciplinary phenomena before the EQ rupture^18^. This physical model is termed “pressure stimulated polarization currents” (PSPC) model^11,12,19,20^ and could be summarized as follows: In the Earth, electric dipoles are always present^19^ due to lattice imperfections (point and linear defects) in the ionic constituents of rocks and exhibit initially random orientations at the future focal region of an EQ, where the stress, $\sigma$, starts to gradually increase. This is called stage A. When this stress accumulation achieves a critical value, the electric dipoles exhibit a cooperative orientation resulting in the emission of a SES activity (cf. cooperativity is a hallmark of criticality^21^). This is called stage B. Uyeda et al.^22^ mentioned that the PSPC model is unique in the following sense: In the PSPC model the SES is generated spontaneously during the gradual increase of stress, while other models that have been proposed for the explanation of the SES generation require a sudden change of stress such as microfracturing. Uyeda et al.^22^ also explained that a direct observation of the spontaneous SES emission cannot be verified in view of the following: laboratory measurements are admittedly hard to perform because one would need to run an electrically well insulated stress machine for a long time with extremely low stress rates until PSPC flows.

The criticality of SES activities has been ascertained by employing natural time analysis (NTA)^23–25^, which has been introduced in 2001^26^ based on a new concept of time termed natural time. NTA enables the uncovering of hidden properties in time series of complex systems and can identify when the system approaches the critical point (for EQs the mainshock occurrence is considered the new phase)^3,27^.

We note that fluctuations are the dominant feature of criticality^28,29^ and close enough to the critical point they become as important as the mean. In particular, the fluctuations of the entropy change under time reversal computed by Eq. (4) are important in order to identify the approach to criticality for major EQs like the M9 Tohoku EQ in Japan in 2011^30^ and the M8.2 EQ in Mexico on 7 September 2017^31^ (see also Chapters 8.2 and 8.4 of Ref.^27^).

Although the stress might be accumulating throughout the entire EQ preparation, it is our purpose here to identify a precursor when the system approaches the critical stage that exhibits different stress accumulation characteristics from those in the long term background.

## Results

Here, we follow the new concept of time introduced in 2001, termed natural time, in which the entropy *S* changes to $S_-$ upon reversing the time arrow (see “Methods”). Then a new complexity measure $\Lambda$ is defined, which quantifies the fluctuations of the quantity $\Delta S \equiv S-S_{-}$ when analysing the earthquake data. We then find that such a procedure identifies when the accumulation of stresses occurs well before the occurrence of major EQs.

**Figure 1:**
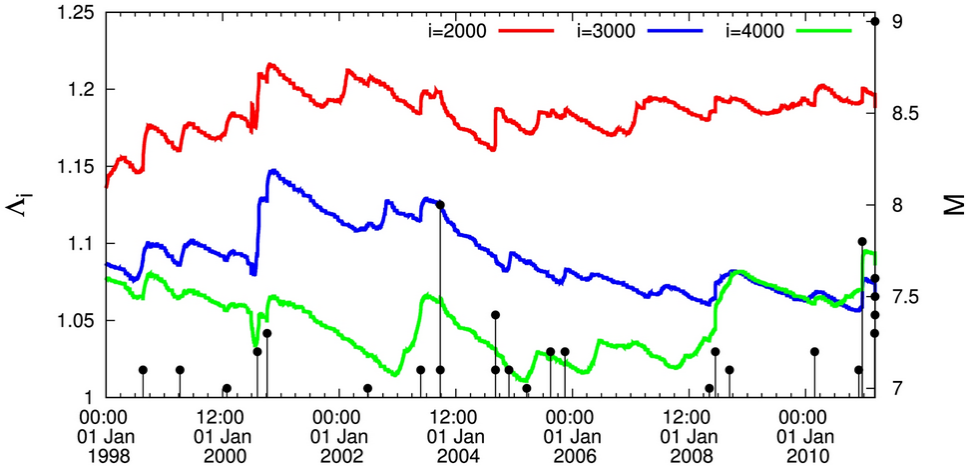
The complexity measure $\Lambda _i$ for various scales *i* = 2000 (red), 3000 (blue), and 4000 (green) versus the conventional time from 1 January 1998 until the *M*9 Tohoku EQ.

**Figure 2:**
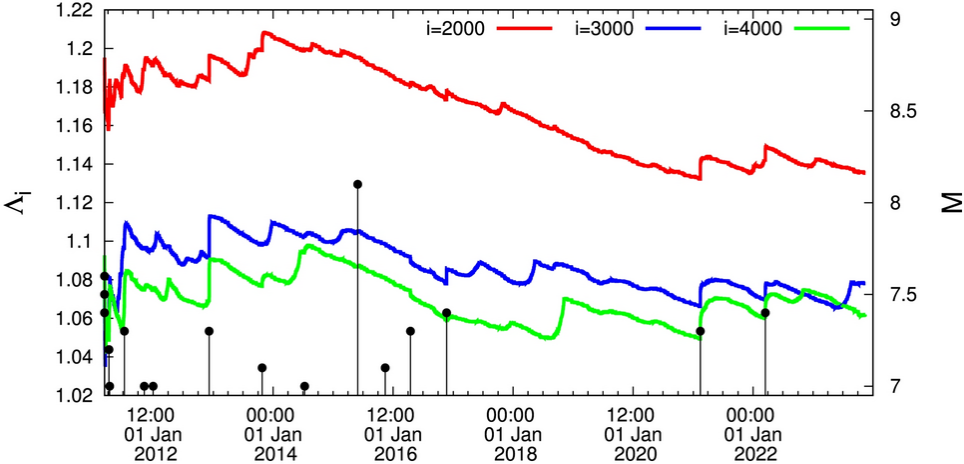
The complexity measure $\Lambda _i$ for various scales *i* = 2000 (red), 3000 (blue), and 4000 (green) versus the conventional time from 15:00 LT on 11 March 2011 until 15 November 2023. The strongest EQ during this period is the Ogasawara EQ, see the text.

**Figure 3:**
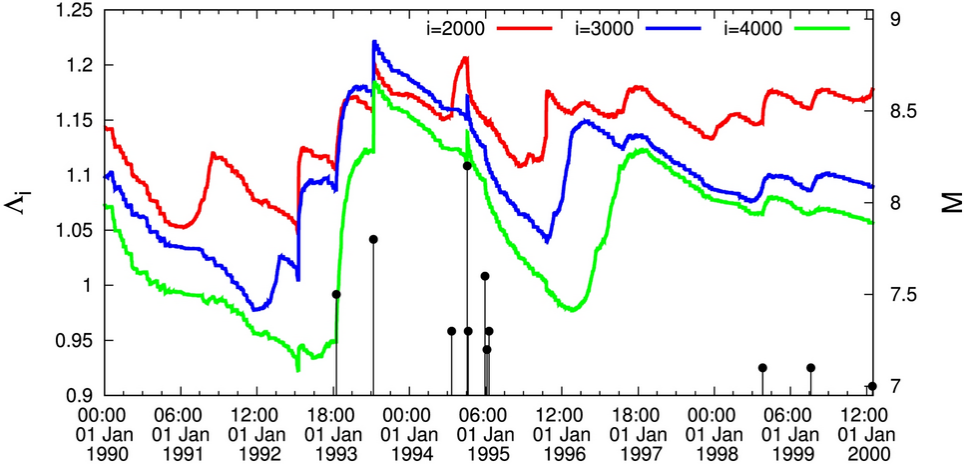
The complexity measure $\Lambda _i$ for various scales *i* = 2000 (red), 3000 (blue), and 4000 (green) versus the conventional time from 1 January 1990 until 1 February 2000.

The results from Japan concerning the study of $\Lambda _i$ are plotted in Figs. 1, 2, and 3 by starting the computation from 1 January 1984 for the scales *i* = 2000, 3000, and 4000 events. After a careful inspection of these figures the following comments are now in order:

### Results from 1 January 1998 until the *M*9 Tohoku EQ occurrence on 11 March 2011

During almost a decade, i.e., during the period from 1 January 1998 until the *M*7.2 EQ on 14 June 2008, there exists no intersection between the curves of the three scales $i=2000$, 3000, and 4000 events since the scale $i=2000$ events lies in the highest level, the scale $i=3000$ events in the middle level and the scale $i=4000$ events in the lowest level. Approximately, from the latter date the curve of the scale $i=4000$ events shows a clear increase, thus finally almost overlapping the curve of the scale $i=3000$ events until almost 5 August 2010. From thereon, however, the curve corresponding to $i=4000$ events exceeds the one of 3000 events (cf. at this date the two curves intersect) and subsequently it exhibits an abrupt increase upon the occurrence of the *M*7.8 EQ on 22 December 2010 in southern Japan at $27.05^{\circ }$ N $143.94^{\circ }$ E, which constitutes an evident intersection. Almost 2$\frac{1}{2}$ months after the completion of this intersection the *M*9 EQ occurred.

Remarkably, on this date (22 December 2010) of the abrupt increase of $\Lambda _i$ additional facts are observed: The abrupt increase conforms to the seminal work by Lifshitz and Slyozov^32^ and independently by Wagner^33^ (LSW) for phase transitions showing that the characteristic size of the minority phase droplets exhibits a scaling behavior in which time (*t*) growth has the form $A (t - t_0)^{1/3}$. It was found that the increase $\Delta \Lambda _i$ of $\Lambda _i$ follows the latter form and that the prefactors *A* are proportional to the scale *i*, while the exponent (1/3) is independent of *i*^30^. Furthermore, the Tsallis^34^ entropic index *q* exhibits a simultaneous increase with the same exponent (1/3)^30^.

In addition, a minimum $\Delta S_{min}$ of the entropy change $\Delta S$ of seismicity in the entire Japanese region under time reversal was identified by Sarlis et al.^35^, who also showed that the probability to obtain such a minimum by chance is approximately 3% thus demonstrating that it is statistically significant. The robustness of the appearance of $\Delta S_{min}$ on 22 December 2010 upon changing the EQ depth, the EQ magnitude threshold, and the size of the area investigated has been documented^35^. Such a minimum is of precursory nature, signaling that a large EQ is impending according to the NTA of the Olami-Feder-Christensen (OFC) model for earthquakes^36^, which is probably^37^ the most studied non-conservative self-organized criticality (SOC) model, originated by a simplification of the Burridge and Knopoff spring-block model^38^. In the OFC model, NTA showed that $\Delta S$ exhibits a clear minimum^3^ before a large avalanche, which corresponds to a large EQ.

Finally, studying the fluctuations $\beta$ of $\kappa _1$ of seismicity in the entire Japanese region N$_{25}^{46}$E$_{125}^{148}$ versus the conventional time from 1 January 1984 until the Tohoku EQ occurrence on 11 March 2011, we find^39^ a large fluctuation of $\beta$ upon the occurrence of the *M*7.8 EQ on 22 December 2010. This finding was also checked for several scales from $i =$ 150 to 500 events, which also revealed the following^39^: upon increasing *i* it is observed (see Figs. 2b and 4e of Ref.^40^) that the increase $\Delta \beta _i$ of the $\beta _i$ fluctuation on 22 December 2010 becomes distinctly larger—obeying the interrelation $\Delta \beta _i = 0.5 \ln (i/114.3)$—which does not happen (see Fig. 4a-d of Ref.^40^) for the increases in the $\beta$ fluctuations upon the occurrences of all other shallow EQs in Japan of magnitude 7.6 or larger during the period from 1 January 1984 to the time of the *M*9 Tohoku EQ. This interrelation $\Delta \beta _i = 0.5 \ln (i/114.3)$, see Fig. 2g,h of Ref.^39^, has a functional form strikingly reminiscent of the one discussed by Penrose et al.^41^ in computer simulations of phase separation kinetics using the ideas of Lifshitz and Slyozov^32^, see their Eq. (33) which is also due to Lifshitz and Slyozov. Hence, the $\beta$ fluctuation on 22 December 2010 accompanying the minimum $\Delta S_{min}$ is unique.

### Results from 15:00 LT on 11 March 2011 until now

During this period, a $M_w$7.9 EQ occurred beneath the Ogasawara (Bonin) Islands on 30 May 2015 as depicted in Fig. 2. It occurred at 680 km depth in an area without any known historical seismicity and caused significant shaking over a broad area of Japan at epicentral distances in the range 1000–2000 km. It was the first EQ felt in every Japanese prefecture since intensity observations began in 1884. This is the deepest EQ ever detected (https://www.nationalgeographic.com/science/article/deepest-earthquake-ever-detected-struck-467-miles-beneath-japan) and was also noted^42^ that globally, this is the deepest (680 km centroid depth) event with $M_w>$ 7.8 in the seismological records.

The Ogasawara EQ has not been followed by an appreciably stronger EQ in contrast to the *M*7.8 Chichi-jima shallow EQ which occurred also at Bonin islands at $27.05^{\circ }$N $143.94^{\circ }$E on 22 December 2010, almost three months before the *M*9 Tohoku EQ. This could be understood as follows^43^: Upon the occurrence of the Chichi-jima EQ the following facts have been observed: First, according to Ref.^30^ the complexity measures $\Lambda _{2000}$, $\Lambda _{3000}$ and $\Lambda _{4000}$, i.e., the $\Lambda _i$ values at the natural time window lengths (scales) *i* = 2000, 3000 and 4000 events, respectively, show a strong abrupt increase $\Delta \Lambda _i$ in Fig. 7 of Ref.^30^ on 22 December 2010 and just after the EQ occurrence exhibiting a scaling behavior of the form $\Delta \Lambda _i = A (t - t_0)^c$ (where the exponent *c* is very close to 1/3 and $t_0$ is approximately 0.2 days after the *M*7.8 EQ occurrence), which conforms to LSW. Second, the order parameter fluctuations showed a unique change^39^, i.e., an increase $\Delta \beta _i$, which exhibits a functional form consistent with the LSW theory and the subsequent work of Penrose et al.^41^ obeying the interrelation $\Delta \beta _i=0.5\ln (i/114.3)$, see Fig. 2g,h of Ref.^39^. Such a behavior has not been observed along with the occurrence of either the Ogasawara EQ or any other shallow EQs in Japan of magnitude 7.6 or larger during the period from 1 January 1984 to the time of the *M*9 Tohoku EQ^39^ (including also the EQ that occurred on 1 January 2024 discussed later).

**Figure 4:**
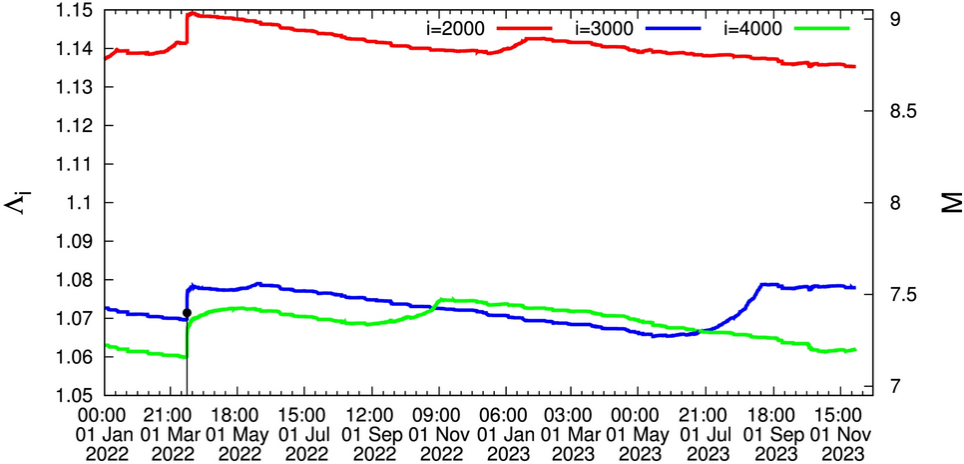
The complexity measure $\Lambda _i$ for various scales *i* = 2000 (red), 3000 (blue), and 4000 (green) versus the conventional time from 1 January 2022 until 15 November 2023.

**Figure 5:**
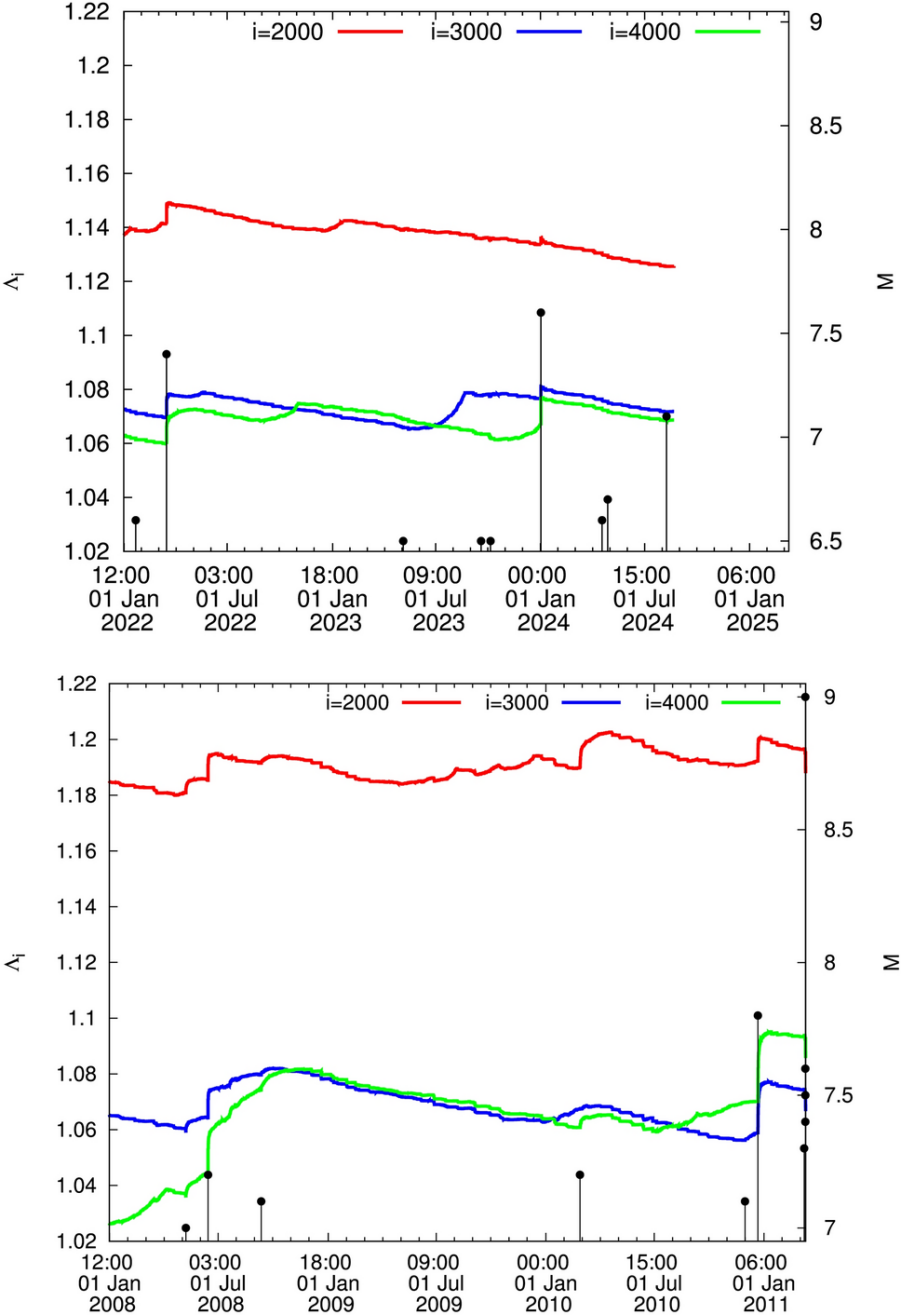
The complexity measure $\Lambda _i$ for various scales $i=2000$ (red), 3000 (blue), and 4000 (green) before the *M*7.6 EQ on the west coast of Japan on 1 January 2024 (upper panel) and the *M*9 Tohoku EQ on 11 March 2011 (lower panel).

An additional important fact is the following: On 27 October 2022, the curve corresponding to the scale $i=4000$ events (green) intersects the one for the scale $i=3000$ (blue), but the latter on 27 June 2023 recovers, see Fig. 4 (we shall return to this important fact later on). This phenomenon has been followed very carefully -since it started as described in Ref.^44^- compared to the one that preceded the *M*9 Tohoku EQ (Fig. 5).

### Results from 1 January 1990 until 1 February 2000

In the relevant plot (Fig. 3), we observe that mostly the curve corresponding to the scale $i=2000$ events lies in the highest level, the curve $i=3000$ events in the middle and the curve $i=4000$ events in the lowest level. There exists, however, the following interesting intersection: Around 8 March 1993 the curve $i=3000$ events jumps to the highest level and remains so until 24 July 1994; subsequently the curve $i=2000$ events returns to the highest level and after that a *M*8.2 EQ occurs on 4 October 1994 (cf., almost 2$\frac{1}{2}$ months after the completion of the intersection between the scale *i* = 3000 events with that of *i* = 2000 events). This is an additional case where a major EQ happens after the detection of an intersection of $\Lambda _i$ curves.

## Discussion

An EQ of JMA magnitude *M* = 7.6 (USGS reported $M_w=7.5$, see, e.g., https://earthquake.usgs.gov/earthquakes/eventpage/us6000m0xl) with epicenter at $37.50^{\circ }$N $137.27^{\circ }$E occurred on the west coast of Japan on 1 January 2024, i.e., almost 3$\frac{1}{2}$ weeks after drawing attention in Ref.^44^ to the important fact focused on the phenomenon described in the last lines of the section describing the results from 15:00 LT on 11 March 2011 until now along with Fig. 4. Referring to the intersection mentioned there, i.e., the curve corresponding to the scale *i* = 4000 events (green) intersects the one for the scale *i* = 3000 (blue), the following comments are now in order: First, the two EQs of magnitude close to *M*8, i.e., the 2003 Tokachi EQ (see Fig. 1) and the 2015 Ogasawara EQ (see Fig. 2), have not been preceded by an intersection (see also Fig. 2 where the green curve approaches -but does not intersect- the blue curve). Second, concerning the *M*8.2 EQ in 1994 -exceeding the aforementioned two EQs of magnitude close to *M*8- there exists an intersection, however, since the curve $i=3000$ events in Fig. 3 jumps to the highest level and an intersection occurs with the curve $i=2000$ events (red) around 8 March 1993. In other words, before 27 October 2022 the only intersection between the curves corresponding to the scales $i=4000$ and $i=3000$ events was observed before the Tohoku *M*9 EQ, see the section describing the Results from 1 January 1998 until the *M*9 Tohoku EQ on 11 March 2011. Thus the phenomenon emerged in Fig. 4 has only appeared before the Tohoku *M*9 EQ as can be visualized in the lower panel of Fig. 5—which is just an excerpt of Fig. 1—showing the following sequence before the Tohoku mainshock: (a)for several months (i.e., approximately 14.5 months from 25 October 2008 to 10 January 2010) the $i=4000$ events curve slightly exceeded the $i=3000$ curve (which actually occurred in the aforementioned 2023 case) and (b)subsequently the $i=3000$ curve recovered for approximately 7 months (from 10 January 2010 to 5 August 2010). Then, a clear intersection occurs on around 5 August 2010 and the $i=4000$ events curve starts to increase more rapidly until 22 December 2010 when a *M*7.8 EQ occurred. Almost two weeks later anomalous variations of the Earth’s magnetic field mainly in the z-component appeared^45^, which in reality reflects that an SES activity that accompanies this magnetic field^15,16^ also started (with a duration of around 10 days) and almost two months later the *M*9 Tohoku mainshock occurred. Notably $\Lambda _{4000}$ exceeded by more than 0.02 $\Lambda _{3000}$ at the time of occurrence of the *M*9 Tohoku EQ.

We also investigated the statistical significance of the phenomenon observed during this 219-day period (5 August 2010 to 11 March 2011) by randomly shuffling the EQ magnitudes from 1 January 1983 to 31 December 2023 and repeating the $\Lambda _i$ calculations. We found that $\Lambda _{4000}$ exceeded by more than 0.01 $\Lambda _{3000}$ at the occurrence time of the *M*9 EQ being previously larger than $\Lambda _{3000}$ for a period smaller than 219 days only in approximately 3% of the 500 synthetic catalogs investigated. Hence, the fact that $\Lambda _{4000}$ exceeded $\Lambda _{3000}$ 219 days before the *M*9 Tohoku EQ cannot be attributed to chance.

**Figure 6:**
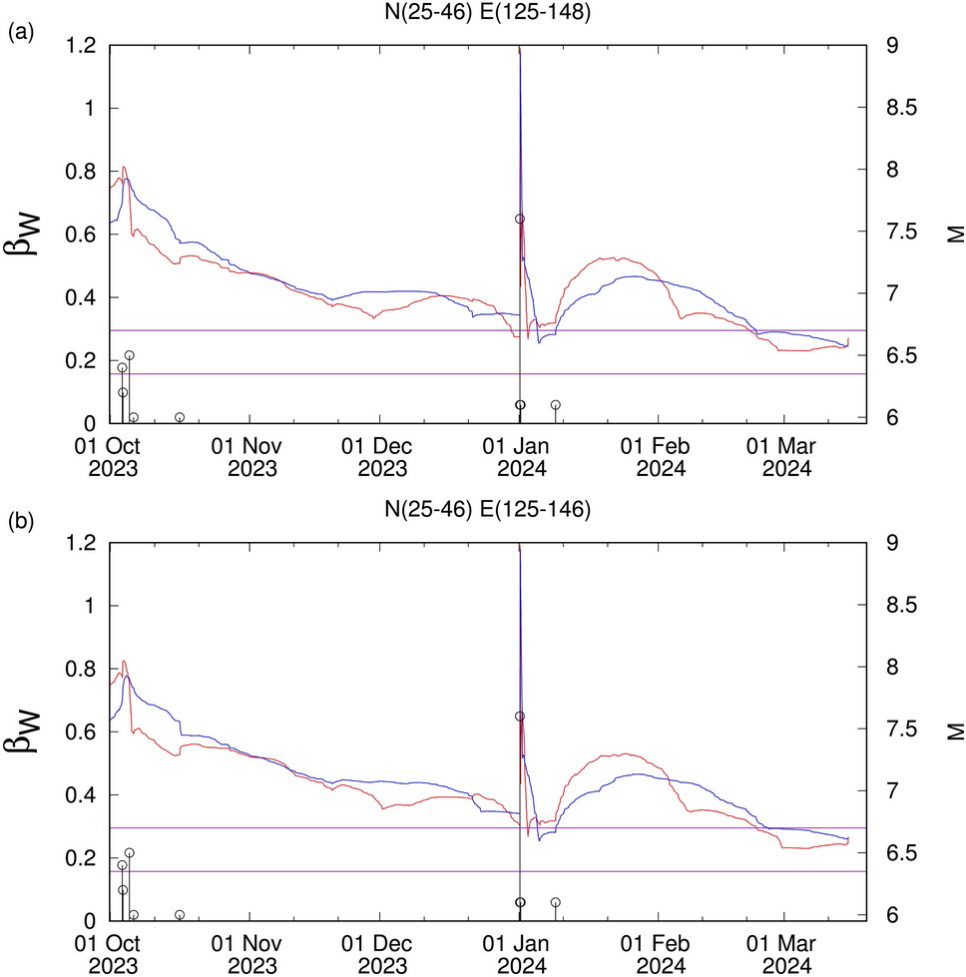
The variability $\beta _W$ for *W* = 200 (red) and 300 (blue) versus the conventional time for (**a**) the area $\hbox {N}^{46}_{25}$ $\hbox {E}^{148}_{125}$ and (**b**) the area $\hbox {N}^{46}_{25}$ $\hbox {E}^{146}_{125}$ since 1 October 2023 until 16 March 2024.

The aforementioned comments shed more light on why the phenomenon in 2023 -depicted in Figs. 4 and 5 (upper panel)- has been, followed very carefully as mentioned in Ref.^44^ by comparing to the one that preceded the *M*9 Tohoku EQ. Furthermore, we note that a minimum $\beta _{W,min}$ of the order parameter fluctuations was found on 3 January 2024 as depicted in Fig. 6 (for the interpretation of these results see Ref.^46^).

We now proceed to the estimation of the statistical significance of the observed phenomenon. As mentioned above on 8 March 1993, i.e., 19 months before the East-Off Hokaido *M*8.2 EQ on 4 October 1994, $\Lambda _{3000}$ exceeded $\Lambda _{2000}$ for the first time. A similar phenomenon concerning $\Lambda _{4000}$ exceeding $\Lambda _{3000}$ occurred on 14 June 2008, i.e., 32 months before the *M*9 Tohoku EQ on 11 March 2011, see the section describing the results from 1 January 1998 until the *M*9 Tohoku EQ on 11 March 2011. Thus, assuming that an alarm is set ON when such intersections occur, we find that for the time period from 1 January 1990 to 1 January 2022 consisting of 384 months the probability to have the alarm ON is $p_{ON}=(19+32)/384=13.28$%. Obviously, the *p*-value to hit by chance both EQs of magnitude *M*8.2 or larger is $p=p_{ON}^2=1.76$%, which points to statistical significance of the phenomenon observed. We clarify that the present calculation of the statistical significance does not include the period depicted in Fig. 4 because the intersection displayed after 1 January 2022 is still under investigation.

Here, we shed light on parameter selection and in particular about the physical reasons that led us to the selection of the scales *i* = 2000, 3000, and 4000 events. We first recall that setting the threshold $M=3.5$ to assure data completeness, we have 47,204 EQs in the study area N$_{25}^{46}$E$_{125}^{148}$ from 1984 to the time of occurrence of Tohoku EQ^40^, which reflects that on the average around 150 EQs occur per month. Second, the fluctuations of the order parameter $\kappa _1$ of seismicity exhibit a minimum $\beta _{min}$ when^47^ an SES activity starts having a lead time ranging from a few weeks up to around $5\frac{1}{2}$ months (with an average value of around a few months or so)^3^. A detailed study^46^ has shown that just before the initiation of the SES activity, the temporal correlations between EQ magnitudes exhibit an anticorrelated behavior^46^ while after this initiation long range correlations prevail. In other words, a significant change in the temporal correlations between EQ magnitudes occurs when comparing the two periods before and just after the initiation of an SES activity. It is this important change that can be captured by the time evolution of $\Delta S_i$ and $\Lambda _i$. Note that the scale of $i \sim 1000$ events is of the order of the number of $M\ge 3.5$ seismic events that occur during a period around the maximum lead time of SES activities. Hence, in our present analysis, in order to first verify the recording of the minimum of $\beta _{min}$ and the subsequent appearance of the initiation of the SES activity (so that to capture the aforementioned important change of EQ temporal correlations) one should use a scale exceeding the $i \sim 1000$ events.

A major EQ does *not* occur alone. In particular, it may be preceded by a foreshock as for example the case of the Tohoku *M*9 EQ in 2011 in which a *M*7.3 foreshock occurred almost 2 days before the mainschock, i.e., on 9 March 2011 at $143.28^{\circ }$E $38.33^{\circ }$N. In addition, a major EQ is followed by afterschocks which usually obey the so called Båth law^48^ according to which the magnitude difference between the mainshock and its largest aftershock is approximately 1.2. A few aftershocks may be also appreciably large EQs as for example the *M*7.6EQ that occurred at 15:15 LT on 11 March 2011, i.e., almost half an hour later than the mainshock, and the *M*7.3EQ at 17:18 LT on 7 December 2012 several months after the mainshock. Thus, in order to include in our analysis the aforementioned important change of the temporal correlations between EQ magnitudes associated with the preceding SES activities of both the relevant foreshocks and aftershocks, we should investigate the scales $i=2000$, 3000, and 4000 events.

**Figure 7:**
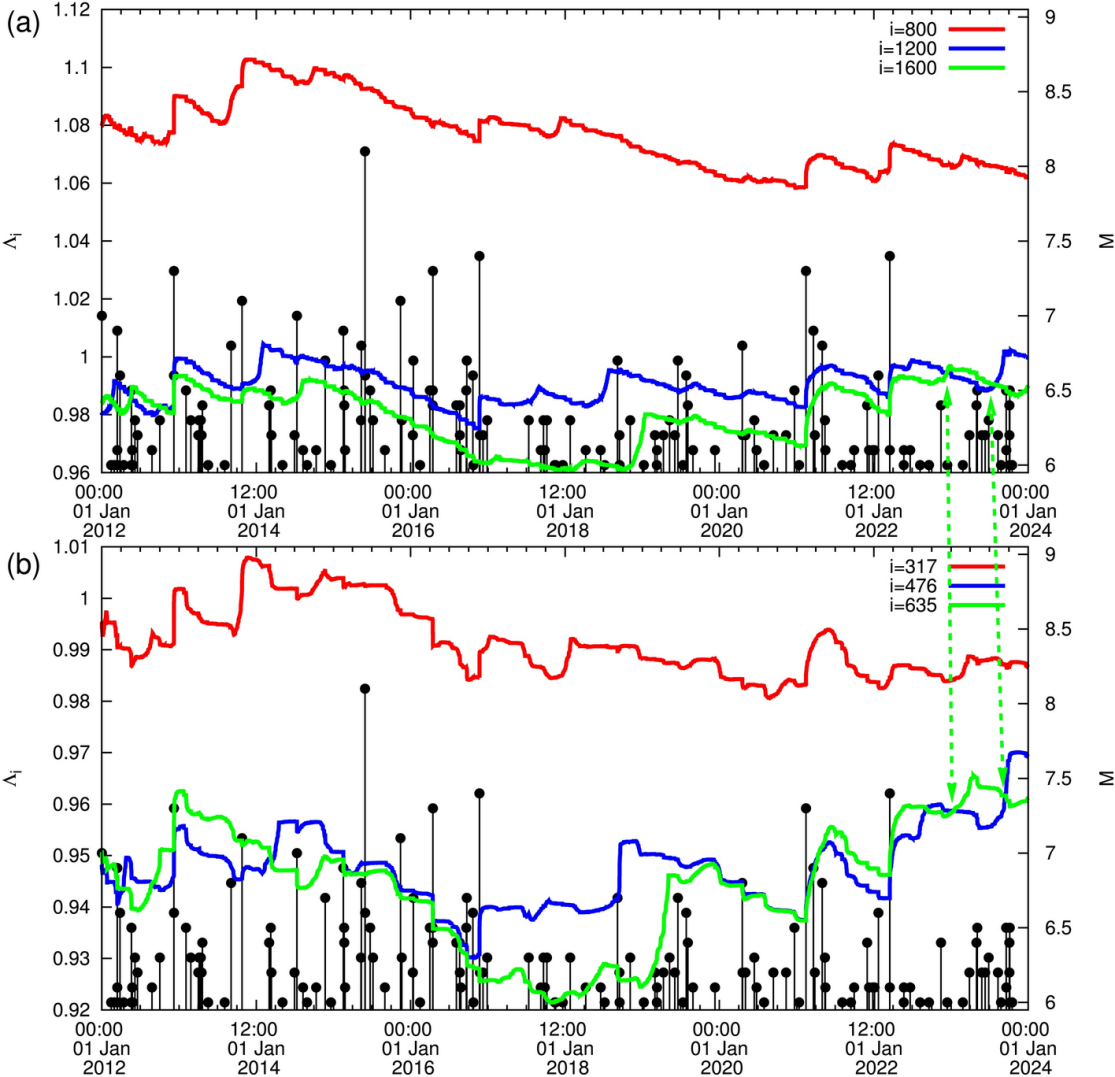
The complexity measure $\Lambda _i$ for various scales when considering (**a**) $M_c$ = 4.0 for *i* = 800 (red), 1200 (blue), and 1600 (green) and (**b**) $M_c$ = 4.5 for $i=317$ (red), $i=476$ (blue), and 635 (green), versus the conventional time from 1 January 2012 until 1 January 2024. The green dashed arrows project the intersection observed for $M_c$ = 4.5 to the results obtained for $M_c$ = 4.0.

We now comment on the uncertainties and limitations of the proposed approach as well as include potential sources of error and how they might affect the results. Returning to the important fact mentioned in the last paragraph of the results from 15:00 LT on 11 March 2011 until now, we recall that in Fig. 4 we used $M_c$ = 3.5 as the cut-off magnitude which can ensure the EQ samples are complete during most of the time. Let us now investigate what happens if $M_c$ will increase to $M_c$ = 4.0 or to $M_c$ = 4.5 shortly after *M*7 class EQs due to aftershock missing and study whether it will potentially affect the complexity measure. Such an $M_c$ increase leads to the results depicted in Fig. 7a which extends from 1 January 2012 until 1 January 2024 upon the occurrence of the *M*7.6 EQ at the western coast of Japan. A careful inspection of this figure shows that, while in Fig. 4 (where $M_c$ = 3.5) it is evident that from 27 October 2022 the curve corresponding to the scale $i=4000$ events intersects the one for the scale $i=3000$, here in Fig. 7a (where $M_c$ = 4.0 resulting^35^ in approximately 2.5 times smaller number of EQs and hence $i=3000$ becomes $i=1200$ and $i=4000$ becomes $i=1600$), such an intersection is delayed to 12 December 2022. In addition, in Fig. 7b (where $M_c$ = 4.5 resulting in approximately 6.3 times smaller number of EQs and hence $i=3000$ becomes $i=476$ and $i=4000$ becomes $i=635$), an intersection can now be recognized on 15 January 2023; the curve corresponding to $i=635$ exceeds the one corresponding to $i=476$ and the latter recovers on 12 September 2023, see the dashed green arrows in Fig. 7. In other words, the $M_c$ value may affect the complexity measure since we have seen that upon using $M_c$ = 3.5 the evident intersection in Fig. 4 (see also Fig. 2) between the scale $i=4000$ events with the scale $i=3000$ events may be retarded (by almost three months), if we increase $M_c$.

**Figure 8:**
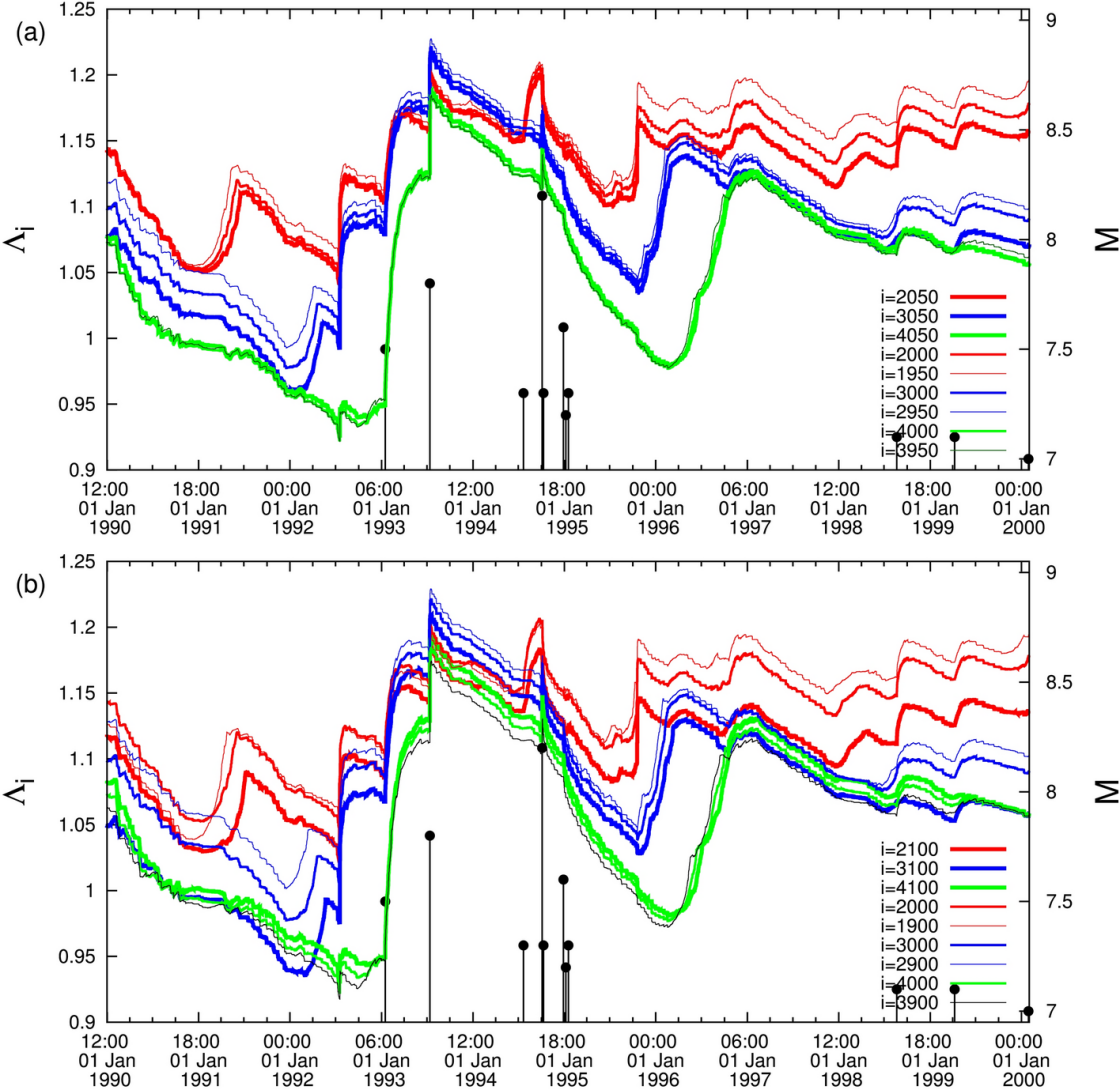
The complexity measure $\Lambda _i$ for various scales *i* versus the conventional time from versus the conventional time from 1 January 1990 until 1 February 2000. Panel (**a**) corresponds to the case when we vary the scales *i* = 2000 (red), 3000 (blue), and 4000 (green) by 50 events, while (**b**) to the case of variation by 100 events.

In Fig. 8, we now present how our results are affected when elaborating the case of the *M*8.2 EQ that occurred in Japan on 4 October 1994 (Fig. 3). We consider two kinds of variation in Fig.8a we increase/decrease *i* by 50 events while in Fig.8b by 100. An inspection of this figure reveals that for the case of Fig. 8a the results remain unchanged while the situation becomes blurred for Fig.8b. Hence, we conclude that a variation of *i* by up to ± 50 events definitely does not affect our proposed method.

A number of methods have appeared for EQ prediction as well as to prepare the population for a big EQ, e.g., see Ref.^49^. For example, both seismic quiescence and seismic activation before large EQs have been repeatedly described in the scientific literature^50^. Another example is an algorithm called M9-MSc, which is based on premonitory seismicity patterns^51^. Furthermore, a promising technique is the so-called region-time-length (RTL) algorithm, which takes into account the epicenter, time, and magnitude of earthquakes, which is an effective technique in detecting seismic quiescence. In particular, Huang and Ding^52^ achieved to reduce the possible ambiguity due to the selection of model parameters in the algorithm and proposed an improved technique of searching for the optimal model parameters. The aforementioned methods are classified as long-term and mid-term EQ prediction methods according to Uyeda et al.^22^ while our efforts based on the discovery of SES and SES activities in 1980s and 1990s together the introduction of the natural time analysis in 2000s (including the present work) to short term EQ prediction methods.

The concept of “natural time” has analogies with the concept of “internal time” by Ilya Prigogine^53^, but there exist also some differences as explained below: The gist of what Prigogine seems to have been after his decades of work seems to be captured in the following 1997 quote: “My colleague Paul Glansdorff and I have investigated the problem as to if the results of near-equilibrium can be extrapolated to those of a far-from-equilibrium situations and have arrived at a surprising conclusion: Contrary to what happens at equilibrium, or near equilibrium, systems far from equilibrium do not conform to any minimum principle that is valid for functions of free energy or entropy production.”^54^ (In place of free energy and entropy functions, Prigogine argues that matter acquires new properties when far from equilibrium in what fluctuations and bifurcations are the norm). On the other hand, in natural time analysis both the minimum $\Delta S_{min}$ and maximum of the fluctuations of $\Delta S$ appear far from equilibrium and in particular when approaching (i.e., a few months before) the EQ occurrence. For example, in the section Results from 1 January 1998 until the *M*9 Tohoku EQ occurrence on 11 March 2011 the following facts have been observed: a minimum of $\Delta S_{min}$ of the entropy change $\Delta S$ of seismicity in the entire Japanese region under time reversal was identified by Sarlis et al.^35^ on 22 December 2010 together with a maximum of the fluctuations of $\Delta S$^30^ (and hence of $\Lambda _i$).

## Summary and conclusions

Studying the evolution of $\Lambda _i$ curves, which quantify the fluctuations of the quantity $\Delta S \equiv S-S_-$, we obtain a procedure that identifies when the accumulation of stress occurs well before major EQs.

**Figure 9:**
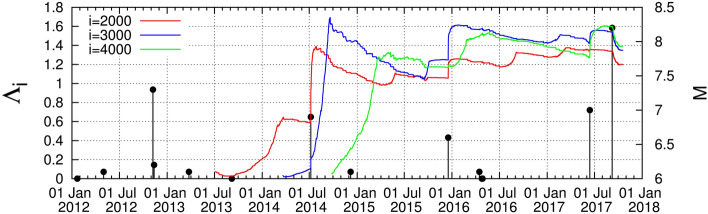
The complexity measure $\Lambda _i$ for various scales *i* = 2000 (red), 3000 (blue), and 4000 (green) versus the conventional time from 1 January 2012 until the *M*8.2 Chiapas EQ in Mexico. Note that the curve corresponding to the scale $i=4000$ exceeds the one of $i=3000$ on 5 July 2017. For details on this calculation see Ref.^31^, see also Sect. 8.4 of Ref.^27^.

In particular, we find for the seismicity of Japan during the last 39 years for various scales *i*( = 2000 to 4000 events), that intersections of these curves occurred before the two strongest EQs (exceeding *M*8), i.e., the *M*9 Tohoku EQ on 11 March 2011 and the East-Off Hokaido *M*8.2 EQ on 4 October 1994. The same phenomenon is ascertained before the deadly Chiapas *M*8.2 EQ in 2017, which is Mexico’s largest EQ in more than a century, as shown in Fig. 9. It is remarkable that all these 3 strong EQs (*M*9, 8.2 and 8.2) occur a few months or so after the completion of their preceding intersections which almost coincides with the lead time of SES activities, hence pointing to the physical mechanism proposed above for the phenomenon observed.

The physical meaning of the phenomenon of the intersection could be understood in the following context: As the system approaches destruction it becomes strongly non-equilibrium acquiring a scale invariant (e.g., multifractal) structure, thus the $\Lambda _i$ values for different scales (*i* = 2000, 3000, 4000 events) may become very close hence the intersection occurs.

## Methods

### The data used

We used the seismic catalog of the Japan Meteorological Agency (JMA) in a similar fashion as in Refs.^40,43,55^ by considering all EQs of magnitude $M\ge 3.5$ to assure data completeness from 1984 within the area $25^{\circ } - 46^{\circ }$N, $125^{\circ } - 148^{\circ }$E. The EQ energy was obtained from the JMA magnitude *M* by converting^56^ to the moment magnitude $M_w$^57^. The $\Lambda _i$ values were computed according to Eq. (4).

### Natural time analysis: background

The motivation and the foundation of natural time analysis take into account the following two key points that have been considered by the first two authors as widely accepted when natural time analysis was proposed in 2001^26^: First, the aspects on the energy of a system forwarded by Max Planck in his Treatise on Thermodynamics in 1945, and second, the theorem on the characteristic functions of probability distributions which Gauss called *Ein Sch*$\ddot{o}$*nes Theorem der Wahrscheinlichkeitsrechnung* (beautiful theorem of probability calculus)^27^.

For a time series comprising *N* events, we define as natural time $\chi _k$ for the occurrence of the *k*-th event the quantity $\chi _k=k/N$^23,26,58^. Hence, we ignore the time intervals between consecutive events, but preserve their order and energy $Q_k$. The evolution of the pair $(\chi _k,p_k)$ is studied, where $p_k=Q_k/\sum _{n=1}^N Q_n$ is the normalized energy for the *k*-th event. Using $\Phi (\omega )=\sum _{k=1}^N p_k \exp (i \omega \chi _k)$ as the characteristic function of $p_k$ for all $\omega \in \mathscr {R}$, the behavior of $\Phi (\omega )$ is studied at $\omega \rightarrow 0$, because all the moments of the distribution of $p_k$ can be estimated from the derivatives $d^m \Phi (\omega ) / d\omega ^m$ (for *m* positive integer) at $\omega \rightarrow 0$. A quantity $\kappa _1$ was defined from the Taylor expansion $\Pi (\omega )= | \Phi (\omega ) |^2 = 1- \kappa _1 \omega ^2 + \kappa _2 \omega ^4 + \ldots$ where

1 $$\begin{aligned} \kappa _1 = \langle \chi ^2 \rangle - \langle \chi \rangle ^2 =\sum _{k=1}^N p_k (\chi _k)^2- \left( \sum _{k=1}^N p_k \chi _k \right) ^2. \end{aligned}$$

A careful study shows^59^ that $\kappa _1$ may be considered as an order parameter of seismicity and was also demonstrated^55^ that the spatiotemporal variations of $\kappa _1$ reveal the epicenters of the EQs of magnitude $M\ge 7.6$. Considering a sliding natural time window of fixed length comprising *W* consecutive events, the variability $\beta _W$ of $\kappa _1$ defined as $\beta _W=\sigma (\kappa _1)/\mu (\kappa _1)$ when for each EQ $e_{i+1}$ in the seismic catalog^40,60^ we calculate the average value $\mu (\kappa _1)$ and the standard deviation $\sigma (\kappa _1)$, the $\kappa _1$ values resulting upon using the previous $\kappa _1$ for the EQs $e_{i-W+1}$ to $e_i$. The fluctuations $\beta _W$ of the order parameter of seismicity exhibit a minimum $\beta _{W,min}$ upon the initiation of an SES activity^47^ upon using a sliding natural time window comprising the number of consecutive events that occur on average within a few months (which is the average lead time of SES activities before major EQs^3^).

The *dynamic* entropy *S* in natural time is given by^25^

2 $$\begin{aligned} S=\langle \chi \ln \chi \rangle - \langle \chi \rangle \ln \langle \chi \rangle , \end{aligned}$$

where $\langle f(\chi ) \rangle =\sum _{k=1}^N p_k f(\chi _k)$ denotes the average value of $f(\chi )$ weighted by $p_k$, i.e., $\langle \chi \ln \chi \rangle = \sum _{k=1}^N p_k (k/N) \ln (k/N)$ and $\langle \chi \rangle = \sum _{k=1}^N p_k (k/N)$. Upon considering^3,61^ the time-reversal $\hat{T}$, i.e., $\hat{T}p_k=p_{N-k+1}$, the entropy obtained by Eq. (2), labelled by $S_-$, is given by

3 $$\begin{aligned} S_-=  \sum _{k=1}^N p_{N-k+1} \frac{k}{N} \ln \left( \frac{k}{N} \right)  -\left( \sum _{k=1}^N p_{N-k+1} \frac{k}{N} \right) \ln \left( \sum _{k=1}^N p_{N-k+1} \frac{k}{N} \right) , \end{aligned}$$

which is different from *S*. Hence, there exists a change $\Delta S \equiv S -S_-$ in natural time under time reversal, thus *S* being time-reversal asymmetric^3,27,61,62^. The calculation of $\Delta S$ is carried out by means of a window of length *i* (=number of successive events), sliding each time by one event, through the whole time series, thus, a new time series comprising successive $\Delta S_i$ values is formed.

The complexity measure $\Lambda _i$ quantifies the fluctuations of the entropy change $\Delta S_i$ under time reversal. It is defined by^3,63^

4 $$\begin{aligned} \Lambda _i = \frac{\sigma (\Delta S_i)}{\sigma (\Delta S_{100})} \end{aligned}$$

where $\sigma (\Delta S_i)$ is the standard deviation of the time series of $\Delta S_i \equiv S_i -(S_-)_i$ and the denominator stands for the standard deviation $\sigma (\Delta S_{100})$ of the time series of $\Delta S_i$ of *i* = 100 events. In other words, it was shown that $\Lambda _i$ quantifies how the statistics of $\Delta S_i$ time series varies upon changing the scale from 100 to another scale *i*. $\Delta S_i$ are of profound importance to study the dynamical evolution of a complex system (see p. 159 of Ref.^3^).

## Data Availability

The authors declare that the data supporting the findings of this study are available within the paper.

## References

[1] Carlson, J. M., Langer, J. S. & Shaw, B. E. Dynamics of earthquake faults. *Rev. Mod. Phys.* **66**, 657–670. 10.1103/RevModPhys.66.657 (1994).

[2] Holliday, J. R. et al. Space-time clustering and correlations of major earthquakes. *Phys. Rev. Lett.* **97**, 238501. 10.1103/PhysRevLett.97.238501 (2006). 17280253 10.1103/PhysRevLett.97.238501

[3] Varotsos, P. A., Sarlis, N. V. & Skordas, E. S. *Natural Time Analysis: The new view of time* (Springer-Verlag, Berlin Heidelberg, Precursory Seismic Electric Signals, 2011).

[4] Huang, Q. Seismicity changes prior to the Ms8.0 Wenchuan earthquake in Sichuan, China. *Geophys. Res. Lett.* **35**, L23308. 10.1029/2008GL036270 (2008).

[5] Huang, Q. Retrospective investigation of geophysical data possibly associated with the Ms8.0 Wenchuan earthquake in Sichuan, China . *J. Asian Earth Sci.* **41**, 421–427. 10.1016/j.jseaes.2010.05.014 (2011).

[6] Telesca, L. & Lovallo, M. Non-uniform scaling features in central Italy seismicity: A non-linear approach in investigating seismic patterns and detection of possible earthquake precursors. *Geophys. Res. Lett.* **36**, L01308. 10.1029/2008GL036247 (2009).

[7] Lennartz, S., Livina, V. N., Bunde, A. & Havlin, S. Long-term memory in earthquakes and the distribution of interoccurrence times. *EPL* **81**, 69001. 10.1209/0295-5075/81/69001 (2008).

[8] Lennartz, S., Bunde, A. & Turcotte, D. L. Modelling seismic catalogues by cascade models: Do we need long-term magnitude correlations?. *Geophys. J. Int.* **184**, 1214–1222. 10.1111/j.1365-246X.2010.04902.x (2011).

[9] Rundle, J. B. et al. Probabilities for large events in driven threshold systems. *Phys. Rev. E* **86**, 021106. 10.1103/PhysRevE.86.021106 (2012). 10.1103/PhysRevE.86.021106 23005722

[10] Turcotte, D. L. *Fractals and Chaos in Geology and Geophysics* (Cambridge University Press, Cambridge, 1997), 2nd edn.

[11] Varotsos, P. & Alexopoulos, K. Physical Properties of the variations of the electric field of the Earth preceding earthquakes. *I. Tectonophysics* **110**, 73–98. 10.1016/0040-1951(84)90059-3 (1984).

[12] Varotsos, P. & Alexopoulos, K. Physical Properties of the variations of the electric field of the Earth preceding earthquakes. *II. Tectonophysics* **110**, 99–125. 10.1016/0040-1951(84)90060-X (1984).

[13] Varotsos, P., Alexopoulos, K., Nomicos, K. & Lazaridou, M. Earthquake prediction and electric signals. *Nature (London)* **322**, 120. 10.1038/322120a0 (1986).

[14] Varotsos, P. & Lazaridou, M. Latest aspects of earthquake prediction in Greece based on Seismic Electric Signals. *Tectonophysics* **188**, 321–347. 10.1016/0040-1951(91)90462-2 (1991).

[15] Varotsos, P. A., Sarlis, N. V. & Skordas, E. S. Electric fields that “arrive’’ before the time derivative of the magnetic field prior to major earthquakes. *Phys. Rev. Lett.* **91**, 148501. 10.1103/PhysRevLett.91.148501 (2003). 14611563 10.1103/PhysRevLett.91.148501

[16] Sarlis, N. & Varotsos, P. Magnetic field near the outcrop of an almost horizontal conductive sheet. *J. Geodynamics* **33**, 463–476. 10.1016/S0264-3707(02)00008-X (2002).

[17] Varotsos, P. *The Physics of Seismic Electric Signals* (TERRAPUB, Tokyo, 2005).

[18] Varotsos, P. A., Sarlis, N. V. & Skordas, E. S. Phenomena preceding major earthquakes interconnected through a physical model. *Ann. Geophys.* **37**, 315–324. 10.5194/angeo-37-315-2019 (2019).

[19] Varotsos, P. & Alexopoulos, K. *Thermodynamics of Point Defects and their Relation with Bulk Properties* (North Holland, Amsterdam, 1986).

[20] Varotsos, P., Alexopoulos, K. & Lazaridou, M. Latest aspects of earthquake prediction in Greece based on Seismic Electric Signals. *II. Tectonophysics* **224**, 1–37. 10.1016/0040-1951(93)90055-O (1993).

[21] Stanley, H. E. Scaling, universality, and renormalization: Three pillars of modern critical phenomena. *Rev. Mod. Phys.* **71**, S358–S366. 10.1103/RevModPhys.71.S358 (1999).

[22] Uyeda, S., Nagao, T. & Kamogawa, M. Short-term earthquake prediction: Current status of seismo-electromagnetics. *Tectonophysics* **470**, 205–213. 10.1016/j.tecto.2008.07.019 (2009).

[23] Varotsos, P. A., Sarlis, N. V. & Skordas, E. S. Long-range correlations in the electric signals that precede rupture. *Phys. Rev. E* **66**, 011902. 10.1103/physreve.66.011902 (2002). 10.1103/PhysRevE.66.011902 12241379

[24] Varotsos, P. A., Sarlis, N. V. & Skordas, E. S. Long-range correlations in the electric signals the precede rupture: Further investigations. *Phys. Rev. E* **67**, 021109. 10.1103/PhysRevE.67.021109 (2003). 10.1103/PhysRevE.67.021109 12636655

[25] Varotsos, P. A., Sarlis, N. V. & Skordas, E. S. Attempt to distinguish electric signals of a dichotomous nature. *Phys. Rev. E* **68**, 031106. 10.1103/PhysRevE.68.031106 (2003). 10.1103/PhysRevE.68.031106 14524749

[26] Varotsos, P. A., Sarlis, N. V. & Skordas, E. S. Spatio-temporal complexity aspects on the interrelation between seismic electric signals and seismicity. *Pract. Athens Acad.* **76**, 294–321. http://physlab.phys.uoa.gr/org/pdf/p3.pdf (2001).

[27] Varotsos, P. A., Sarlis, N. V. & Skordas, E. S. *Natural Time Analysis: The new view of time, Part II. Advances in Disaster Prediction using Complex Systems* (Springer Nature Switzerland AG, Cham, 2023).

[28] Riquelme-Galván, M. & Robledo, A. Dual characterization of critical fluctuations: Density functional theory & nonlinear dynamics close to a tangent bifurcation. *Eur. Phys. J. Spec. Top.* **226**, 433–442. 10.1140/epjst/e2016-60268-0 (2017).

[29] Varotsos, P. A. et al. Improving the Estimation of the Occurrence Time of an Impending Major Earthquake Using the Entropy Change of Seismicity in Natural Time Analysis. *Geosciences* **13**, 222. 10.3390/geosciences13080222 (2023).

[30] Varotsos, P. A., Sarlis, N. V. & Skordas, E. S. Tsallis Entropy Index q and the Complexity Measure of Seismicity in Natural Time under Time Reversal before the M9 Tohoku Earthquake in 2011. *Entropy* **20**, 757. 10.3390/e20100757 (2018). 33265846 10.3390/e20100757 PMC7512320

[31] Ramírez-Rojas, A., Flores-Márquez, E. L., Sarlis, N. V. & Varotsos, P. A. The Complexity Measures Associated with the Fluctuations of the Entropy in Natural Time before the Deadly Mexico M8.2 Earthquake on 7 September 2017. *Entropy* **20**, 477. 10.3390/e20060477 (2018). 10.3390/e20060477 PMC7512995 33265567

[32] Lifshitz, I. & Slyozov, V. The kinetics of precipitation from supersaturated solid solutions. *J. Phys. Chem. Solids* **19**, 35–50. 10.1016/0022-3697(61)90054-3 (1961).

[33] Wagner, C. Theorie der alterung von niederschlägen durch umlösen (ostwald-reifung). *Zeitschrift für Elektrochemie, Berichte der Bunsengesellschaft für physikalische Chemie* **65**, 581–591. 10.1002/bbpc.19610650704 (1961).

[34] Tsallis, C. Possible generalization of Boltzmann-Gibbs statistics. *J. Stat. Phys.* **52**, 479–487. 10.1007/BF01016429 (1988).

[35] Sarlis, N. V., Skordas, E. S. & Varotsos, P. A. A remarkable change of the entropy of seismicity in natural time under time reversal before the super-giant M9 Tohoku earthquake on 11 March 2011. *EPL* **124**, 29001. 10.1209/0295-5075/124/29001 (2018). 10.3390/e20100757 PMC7512320 33265846

[36] Olami, Z., Feder, H. J. S. & Christensen, K. Self-organized criticality in a continuous, nonconservative cellular automaton modeling earthquakes. *Phys. Rev. Lett.* **68**, 1244–1247. 10.1103/physrevlett.68.1244 (1992). 10046116 10.1103/PhysRevLett.68.1244

[37] Ramos, O., Altshuler, E. & Måløy, K. J. Quasiperiodic events in an earthquake model. *Phys. Rev. Lett.* **96**, 098501. 10.1103/physrevlett.96.098501 (2006). 16606323 10.1103/PhysRevLett.96.098501

[38] Burridge, R. & Knopoff, L. Model and theoretical seismicity. *Bull. Seismol. Soc. Am.* **57**, 341–371. 10.1785/BSSA0570030341 (1967).

[39] Varotsos, P. A., Sarlis, N. V. & Skordas, E. S. Natural time analysis: Important changes of the order parameter of seismicity preceding the 2011 M9 Tohoku earthquake in Japan. *EPL* **125**, 69001. 10.1209/0295-5075/125/69001 (2019).

[40] Sarlis, N. V. et al. Minimum of the order parameter fluctuations of seismicity before major earthquakes in Japan. *Proc. Natl. Acad. Sci. U.S.A.* **110**, 13734–13738. 10.1073/pnas.1312740110 (2013). 23918353 10.1073/pnas.1312740110 PMC3752201

[41] Penrose, O., Lebowitz, J. L., Marro, J., Kalos, M. H. & Sur, A. Growth of clusters in a first-order phase transition. *J. Stat. Phys.* **19**, 243–267. 10.1007/BF01011725 (1978).

[42] Ye, L., Lay, T., Zhan, Z., Kanamori, H. & Hao, J.-L. The isolated 680 km deep 30 May 2015 Mw 7.9 Ogasawara (Bonin) Islands earthquake. *Earth Planet. Sci. Lett.* **433**, 169–179. 10.1016/j.epsl.2015.10.049 (2016).

[43] Varotsos, P. A., Sarlis, N. V., Skordas, E. S., Nagao, T. & Kamogawa, M. The unusual case of the ultra-deep 2015 Ogasawara earthquake (M7.9): Natural time analysis. *EPL* **135**, 49002. 10.1209/0295-5075/135/49002 (2021).

[44] Varotsos, P. A., Nagao, T. & Sarlis, N. V. A complexity measure identifying the accumulation of stresses before major earthquakes. arXiv:2312.02900v1 (2023).10.1038/s41598-024-81547-z39730642

[45] Skordas, E. & Sarlis, N. On the anomalous changes of seismicity and geomagnetic field prior to the 2011 9.0 Tohoku earthquake. *J. Asian Earth Sci.* **80**, 161–164. 10.1016/j.jseaes.2013.11.008 (2014).

[46] Varotsos, P. A., Sarlis, N. V. & Skordas, E. S. Study of the temporal correlations in the magnitude time series before major earthquakes in Japan. *J. Geophys. Res.: Space Phys.* **119**, 9192–9206. 10.1002/2014JA020580 (2014).

[47] Varotsos, P. A., Sarlis, N. V., Skordas, E. S. & Lazaridou, M. S. Seismic Electric Signals: An additional fact showing their physical interconnection with seismicity. *Tectonophysics* **589**, 116–125. 10.1016/j.tecto.2012.12.020 (2013).

[48] Båth, M. Lateral inhomogeneities of the upper mantle. *Tectonophysics* **2**, 483–514. 10.1016/0040-1951(65)90003-X (1965).

[49] Ikarashi, A. These labs have prepared for a big earthquake - will it be enough?. *Nature*10.1038/d41586-024-02622-z (2024). 39155315 10.1038/d41586-024-02622-z

[50] Sobolev, G. Seismic quiescence and activation. In Gupta, H. K. (ed.) *Encyclopedia of Solid Earth Geophysics*, 1178–1184 (Springer Netherlands, Dordrecht, 2011). 10.1007/978-90-481-8702-7_185.

[51] Davis, C., Keilis-Borok, V., Kossobokov, V. & Soloviev, A. Advance prediction of the march 11, 2011 great east japan earthquake: A missed opportunity for disaster preparedness. *Int. J. Disaster Risk Reduct.* **1**, 17–32. 10.1016/j.ijdrr.2012.03.001 (2012).

[52] Huang, Q. & Ding, X. Spatiotemporal variations of seismic quiescence prior to the 2011 M 9.0 Tohoku earthquake revealed by an improved region-time-length algorithm. *Bull. Seismol. Soc. Am.* **102**, 1878–1883. 10.1785/0120110343 (2012).

[53] Prigogine, I. *From Being to Becoming: Time and Complexity in the Physical Sciences* (W.H. Freeman and Company, San Francisco, 1980).

[54] Prigogine, I. *The End of Certainty: Time, Chaos, and the New Laws of Nature* (Free Press, New York, 1997) (p. 64).

[55] Sarlis, N. V. et al. Spatiotemporal variations of seismicity before major earthquakes in the Japanese area and their relation with the epicentral locations. *Proc. Natl. Acad. Sci. U.S.A.* **112**, 986–989. 10.1073/pnas.1422893112 (2015). 25548194 10.1073/pnas.1422893112 PMC4313817

[56] Tanaka, H. K., Varotsos, P. A., Sarlis, N. V. & Skordas, E. S. A plausible universal behaviour of earthquakes in the natural time-domain. *Proc. Jpn. Acad. Ser. B Phys. Biol. Sci.* **80**, 283–289. 10.2183/pjab.80.283 (2004).

[57] Kanamori, H. Quantification of earthquakes. *Nature* **271**, 411–414. 10.1038/271411a0 (1978).

[58] Varotsos, P. A., Sarlis, N. V. & Skordas, E. S. Seismic Electric Signals and Seismicity: On a tentative interrelation between their spectral content. *Acta Geophys. Pol.* **50**, 337–354. http://physlab.phys.uoa.gr/org/pdf/d35.pdf (2002).

[59] Varotsos, P. A., Sarlis, N. V., Tanaka, H. K. & Skordas, E. S. Similarity of fluctuations in correlated systems: The case of seismicity. *Phys. Rev. E* **72**, 041103. 10.1103/physreve.72.041103 (2005). 10.1103/PhysRevE.72.041103 16383358

[60] Sarlis, N. V., Skordas, E. S. & Varotsos, P. A. Order parameter fluctuations of seismicity in natural time before and after mainshocks. *EPL* **91**, 59001. 10.1209/0295-5075/91/59001 (2010).

[61] Varotsos, P. A., Sarlis, N. V., Tanaka, H. K. & Skordas, E. S. Some properties of the entropy in the natural time. *Phys. Rev. E* **71**, 032102. 10.1103/physreve.71.032102 (2005). 10.1103/PhysRevE.71.032102 15903469

[62] Varotsos, P. A., Sarlis, N. V., Skordas, E. S. & Lazaridou, M. S. Identifying sudden cardiac death risk and specifying its occurrence time by analyzing electrocardiograms in natural time. *Appl. Phys. Lett.* **91**, 064106. 10.1063/1.2768928 (2007).

[63] Sarlis, N. V., Christopoulos, S.-R.G. & Bemplidaki, M. M. Change of the entropy in natural time under time reversal: Complexity measures upon change of scale. *EPL* **109**, 18002. 10.1209/0295-5075/109/18002 (2015).

